# Increased Prefrontal and Parahippocampal Activation with Reduced Dorsolateral Prefrontal and Insular Cortex Activation to Food Images in Obesity: A Meta-Analysis of fMRI Studies

**DOI:** 10.1371/journal.pone.0060393

**Published:** 2013-04-10

**Authors:** Samantha J. Brooks, Jonathan Cedernaes, Helgi B. Schiöth

**Affiliations:** Department of Neuroscience, Uppsala University, Uppsala, Sweden; Bellvitge Biomedical Research Institute-IDIBELL, Spain

## Abstract

**Background and Objectives:**

Obesity is emerging as the most significant health concern of the twenty-first century. A wealth of neuroimaging data suggest that weight gain might be related to aberrant brain function, particularly in prefrontal cortical regions modulating mesolimbic addictive responses to food. Nevertheless, food addiction is currently a model hotly debated. Here, we conduct a meta-analysis of neuroimaging data, examining the most common functional differences between normal-weight and obese participants in response to food stimuli.

**Data Source:**

We conducted a search using several journal databases and adhered to the ‘Preferred Reporting Items for Systematic Reviews and Meta-analyses’ (PRISMA) method. To this aim, 10 studies were found with a total of 126 obese participants, 129 healthy controls, equaling 184 foci (146 increased, 38 decreased activation) using the Activation Likelihood Estimation (ALE) technique. Out of the 10 studies, 7 investigated neural responses to food versus non-food images.

**Results:**

In response to food images, obese in comparison to healthy weight subjects had increased activation in the left dorsomedial prefrontal cortex, right parahippocampal gyrus, right precentral gyrus and right anterior cingulate cortex, and reduced activation in the left dorsolateral prefrontal cortex and left insular cortex.

**Conclusions:**

Prefrontal cortex areas linked to cognitive evaluation processes, such as evaluation of rewarding stimuli, as well as explicit memory regions, appear most consistently activated in response to images of food in those who are obese. Conversely, a reduced activation in brain regions associated with cognitive control and interoceptive awareness of sensations in the body might indicate a weakened control system, combined with hypo-sensitivity to satiety and discomfort signals after eating in those who are prone to overeat.

## Introduction

An abundance of easy access and exposure to high-energy palatable food, food advertising and increasingly competitive stressful work schedules have all contributed to a change in the way people relate to food, most commonly manifesting as an increase in obesity and binge-eating. Anticipation of food consumption is likely greater with advertising reminders that in Western societies food is in abundance. Obesity affects approximately 500 million adults worldwide according to the World Health Organisation (WHO) [1]. A wealth of neuroimaging data on eating behaviour over the last decade suggests that difficulties in the regulation of food intake may be due to aberrant brain function, particularly in prefrontal cortical regions that modulate mesolimbic reward responses to food, as well as appetitive and somatosensory regions such as the striatum, hypothalamus and insular cortex [2]. Neuroimaging of neural responses in those with obesity or who are overweight has largely been conducted using food images as opposed to actual food ingestion, and a meta-analysis of these studies would provide a better understanding of the neural mechanisms underlying the development of obesity.

Recently, brain imaging data have supported an addiction model of obesity [3], emphasising a disequilibrium between cognitive control and reward sensitivity and the contribution of brain reward circuits to the obesity epidemic [4]. From this perspective, a recent review has described neurobehavioral vulnerability that likely underpins addiction to food in those who are obese, encompassing reduced brain function in regions associated with homeostatic satiety and cognitive inhibition of appetite [5]. However, there are arguments against a model describing an impairment of cognitive control - a central tenet of the addiction model of obesity [6], [7], and that the preliminary evidence applies more to binge eating specifically, with further work necessary to clarify a neurobiological model of food addiction in obesity [8]. It is not currently clear whether the addiction neurobiological model of obesity is accurate, specifically in terms of activation in prefrontal cortex regions.

Thus, in the present study we conduct a meta-analysis of fMRI studies to investigate systematically the most prevalent patterns of neural activation to food stimuli, with a primary focus on images of food in obese humans, to add to the debate on the neurobiological addiction model of obesity. We meta-analyse cross-sectional fMRI studies that compare neural activation in obese versus healthy controls. The Activation Likelihood Estimation (ALE) GingerAle BrainMap method [9], [10] is a contemporary fMRI meta-analysis tool that has been used in various other meta-analyses to quantify data from fMRI studies, e.g.: [11], [12], [13], [14], [15]. Using this method, we present ALE meta-analyses on the neural responses to food stimuli in obese humans. Against the background of previous brain imaging data, specifically in line with the addiction model of obesity, we hypothesise that dorsolateral prefrontal cortex activation will be reduced, and that regions associated with reward evaluation, somatosensory responses and motivation (e.g. orbitofrontal cortex, striatum, insular cortex) will show increased activation to food images.

## Materials and Methods

### Ethics Statement

This work meets all ethical guidelines as set out by the Declaration of Helsinki and Uppsala University, as well as Uppsala Ethical Board.

### Searching Procedure

PubMed, Medline, Ovid, Sciencedirect, Web of Science and Google Scholar were searched, and hand searches were completed of the reference lists from all studies found, between the year 2000 and December 2012. Search terms were: “fMRI AND food” (n = 1413), “Eating AND fMRI” (n = 481), “Binge AND food AND fMRI” (n = 12), “Appetite AND fMRI” (n = 244) and “Obese OR overweight AND fMRI” (n = 1640).

### Inclusion and Exclusion Criteria

Criteria for inclusion were: a) any fMRI studies published up to December 2012, b) case-control studies reporting on fMRI activation to food images, c) cases with overweight or obesity and not other types of eating disorder (e.g. anorexia nervosa, bulimia nervosa binge eating disorder), d) fMRI (functional Magnetic Resonance Imaging) and not other functional or structural brain imaging techniques (e.g., Positron Emission Tomography [PET], Computed Axial Tomography [CAT], Magnetic Resonance Spectroscopy [MRS], single photon emission computed tomography [SPECT]), e) paradigms that used food stimuli and contrasted fMRI BOLD signal changes from food stimuli versus neutral or non-food stimuli, f), original English-language articles, g) published in a peer-reviewed journal, h) fMRI coordinates were reported in either Talairach [16] or Montreal Neurological Institute space; in the latter case converted into Talairach space for this review; i) data from Whole Brain (WB) and not Region of Interest (ROI) analysis, as ROI data can artificially inflate the data [17]. All excluded studies are listed in Table S1.

### Identification, Screening, Eligibility

See Figure S1 for a PRISMA diagram illustrating the study selection steps. See Table S1 for initial search exclusions, Table S2 for exclusions after initial eligibility screening and Table S3 for PRISMA checklist of items. Specifically, we first found 3790 records, but 3307 of these were duplicated records, or studies that did not fit our criteria at all (e.g. genetic studies, studies of adiposity, studies of bacteria etc). Of 483 studies screened as eligible, 407 records were excluded due to the fMRI studies were measuring only healthy controls (n = 158), the papers were reviews/editorials or meta-analyses (n = 55), non-fMRI neuroimaging studies (n = 37), case reports (n = 36), the fMRI studies did not use food images (n = 37), there was no healthy control comparison group (n = 27), or the papers presented case reports (n = 36). Of the remaining 76 fMRI studies assessed for eligibility, 40 were excluded because they reported other eating disorders not obesity (e.g. anorexia nervosa, bulimia nervosa, Prader Willi syndrome) and finally 29 fMRI studies were excluded as they compared obese participants to healthy controls but only using Region of Interest (ROI) analysis (n = 15), or reported no case-control comparison (activation and/or deactivation) data (n = 14). However, out of the 15 ROI studies, three also contained WB analyses that fit our inclusion criteria (see above) and WB case-control comparison data from these three studies were therefore also included. Thus, we meta-analysed a total of 10 fMRI studies conducting whole brain analyses, comparing neural responses to food versus non-food stimuli, in a secondary meta-analysis. For our primary meta-analysis we examined only neural responses to food images, and in this 7 out of the 10 included studies were included.

See Table 1 for study characteristics of the studies included in the primary (n = 7) and secondary (n = 10) meta-analysis.

**Table 1:** Non Region of Interest (ROI) Whole Brain fMRI studies and experiments included in ALE meta-analyses (total study n = 10) comparing food versus non-food *stimuli*.

Author (yr, ref)	Stimulus	Control Condition	No. of Cases			No. Of Controls		Foci	*Brain*
			Age, range orMean (s.d.)/Gender			Age, range or*Mean (s.d.)/* *Gender*		*(activation/* *Deactivation)*	*analysis*
**Bragulat et al.,**	Food odors	Nonappetitive odors	5 f			5 f		15 (6;9)	WB
**(2010) ** [**52**]			31.6 (8.8)			23.4 (1.1)			
**Cornier et al.,**	Food images	Non-food images	19 (ob) 9 m, 10 f			22, 12 m, 10 f		3 (3;0)	WB
**(2009) ** [**53**]			35.5 (5.7)			34.4 (5.1)			
**Davids et al.,**	Food images	Neutral images	22 (ob/ov) 7 m, 15 f			22, 10 m, 12 f			
**(2010) ** [**54**]			13.5 (2.9)/			13.5 (2.3)		10 (0;10)	WB/ROI
**Dimitropoulos et al.,**	Food images	Images of objects	22 (ob) 11 m, 11 f			16, 6 m, 10 f		38 (29;9)	WB
**(2012) ** [**55**]			24.8 (6.7)			24.6 (4.2)			
**Martin et al.,**	Food images	Image of animals	10 (ob) 5 m, 5 f			10 5 m, 5 f		34 (32;2)	WB
(**2010) ** [**23**]			(n/a)			(n/a)			
**Oltmans et al.,**	Food images	Images of objects	10 f			10 f		18 (17;1)	WB/ROI
**(2012) ** [**56**]			20–45			23–45			
***Stice et al.,***	Chocolate milk	Tasteless solution	10 (ob) f			11 f		25 (19;6)	WB
***(2008) *** [***57***]			15.7(0.9)			15.7(0.9)			
**Stice et al.,**	Chocolate milk	Tasteless solution	7 (ob) f			11 f		17 (17;0)	WB
**(2008) ** [**58**]			15.7(0.9)			15.7(0.9)			
**Stice et al.,**	Food images	Food images	9 (ob) f			10 f		1 (0;1)	WB/ROI
**(2010) ** [**59**]		(non-appetising)	21.0 (1.1)			21.1 (1.1)			
**Szalay et al.,**	Sucrose solution	Water	12 (ob) 3 m, 9 f			12 (n/a)		23 (23;0)	WB
**(2012) ** [**60**]			38.3 (4.2)			37.1 (3.8)			
*Total number (total for only food image stimuli studies):*				*126 (99)*			*129 (101)*		
					*184 (121)*				

Studies in bold italic font were included in the second but excluded from the first meta-analysis, which only examined non-ROI studies comparing food versus non-food *images* (total study n = 7).

Abbreviations: ob = obese, ob child = obese children, ov = overweight; n/a = age data not available; f = female; m = male, WB = whole brain, ROI = region of interest.

### Food Stimuli in fMRI Studies

To examine general neural responses to food stimuli in those who were obese or overweight at time of scan versus healthy controls, we chose fMRI studies that used food stimuli (and not, e.g., body image stimuli) in people who were not fasted. The studies that met our inclusion criteria mainly employed *images* of food (7/10 studies). However, there were also a limited number of studies with food stimuli other than images, such as intake, taste and odors of food versus (3/10 studies). Control conditions were: non-food images, images of inedible food, blurred images, images of cars, images of household objects, a fixation cross; or for the non-food images studies: intake of tasteless solution, tasting water or non-appetitive odors. See Table 1. We extracted Talairach coordinates from all included papers, as specified by the GingerALE Activation Likelihood Estimation approach (described below) [17], for regions of increased or decreased activation, referred to hereafter as foci, when comparing obese with healthy control subjects. We ran primary (only food images) and secondary (all types of food stimuli) meta-analyses, and report these separately in the results section.

### fMRI Methods

Functional Magnetic Resonance Imaging (fMRI) is a brain imaging technique that uses Blood Oxygen Level Dependency (BOLD) as an indirect measure of neural activation. For a more detailed description of fMRI, particularly in relation to its clinical potential, see [18].

### Activation Likelihood Estimation (ALE)

ALE is a statistical modeling technique that uses the total foci coordinates reported in each study to build a 3-dimensional Gaussian kernel in order to create a model activation (MA) map for each study. We followed the GingerALE protocol (http://www.brainmap.org/ale/) as devised by Eickhoff et al., [17]. The position of foci can be a consequence of between-study variances, such as the different templates used, or the differences between participants, and as such these two main issues are considered in the parameters of the kernel. This is done by weighting the foci reported by the number of participants in each study. Finally, the MA maps for each study are combined for each separate meta-analysis, creating an experimental ALE map. This is tested against the null hypothesis that there is random variation in relation to the spatial orientation of neural activation for the specific meta-analysis, but that the within-study variation is fixed. A random effects model is employed by the ALE analysis technique, which assumes a higher than chance likelihood of consensus between different experiments, but not in relation to activation variance within each study. The null distribution map is permuted by the number of studies that constitute each meta-analysis. To correct for multiple comparisons, we used a threshold of p<0.05 False Discovery Rate (FDR), and chose a minimum cluster size of 100 mm^3^ in line with a recently published fMRI ALE meta-analysis [19]. We used an anatomical image overlay program called Mango (Creators, Jack Lancaster, Michael Martinez: http://ric.uthscsa.edu/mango) to illustrate the results of our meta-analyses with Talairach coordinates. All of these steps combined help to control for publication bias with regard to reported foci.

GingerALE employs the term “contributing studies”, to describe studies that are located within the boundaries of an ALE cluster. However, this does not discount other studies, which might be located near these boundaries but outside of the cluster, could have also contributed to it.

## Results

### Primary Meta-analysis: Food Images versus Non-food Images

#### Increased activation in obese versus healthy controls

**Figure 1:**
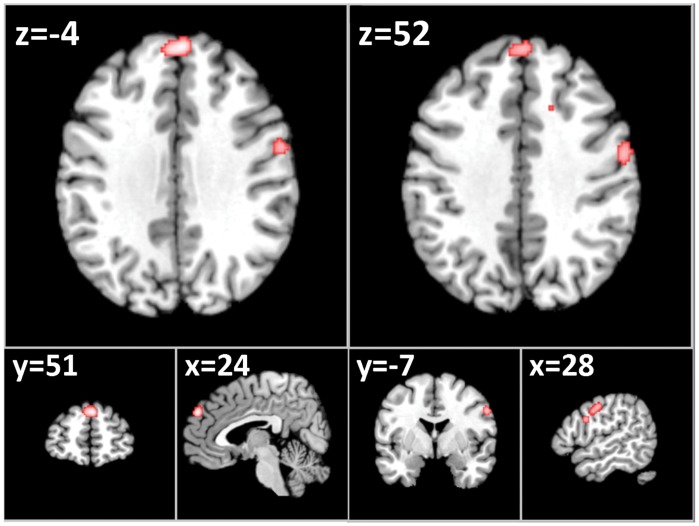
Axial (z), coronal (y), saggital (x) views of increased activation to food images in fMRI studies of obese/overweight (versus healthy controls) excluding Region of Interest Studies - FDR corrected, p<0.05 for multiple comparisons, cluster size >100 **mm^3^.** Left: left dorsomedial prefrontal cortex (dmPFC); right: increased activation in the right precentral gyrus.

In response to food versus non-food images, a total of 5 of the 7 non-ROI studies contrasting food to non-food images contributed to 6 clusters of increased activation after threshold correction (FDR) in the left dorsomedial prefrontal cortex (x = −4, y = 51, z = 24), right precentral gyrus (x = 52, y = −7, z = 28), right parahippocampal gyrus (x = 21, y = −48, z = 1), right inferior frontal gyrus (x = 50, y = 4, z = 16), right superior frontal gyrus (x = 19, y = 15, z = 48) and anterior right cingulate gyrus (x = 12, y = 17, z = 31). See Table 2A and Figure 1.

**Table 2:** Locations of centered Talairach peak coordinates with significant ALE values for, respectively, *increased* (1) and decreased (2) activation in obese/overweight versus control subjects for all non-ROI fMRI studies looking at *food versus non-food image stimuli* in the meta-analysis.

Cluster^a^			Anatomical Label^b^	Peak voxel coordinates^c^			Cluster size	ALE value	Foci	
1.Obese/overweight>Normal-weight controls (5 contributing studies)										
				*x*	*y*	z	(mm^3^)	(×10^−2^)	*n*	*foci*
1		L. Dorsoedial PFC		−4	51	24	928	2.17	2	5
2		R. Precentral Gyrus		52	−7	28	568	1.66	1	4
3		R. Parahipp. Gyrus		21	−48	2	344	1.72	1	2
4		R. Inferior Frontal Gyrus		50	4	16	216	1.65	1	2
5		R. Superior Frontal Gyrus		19	15	48	216	1.67	1	2
6		R. Cingulate Gyrus		12	17	32	200	1.61	2	2
2.Normal−weight controls>Obese/overweight (6 contributing studies)										
				*x*	*y*	*z*	(mm^3^)	(×10^−2^)	*n*	*foci*
1	L. DLPFC			−29	29	36	336	1.47	1	2
2	L. Insula			−43	0	9	272	1.25	1	2

#### Decreased activation in all obese cases versus controls

**Figure 2:**
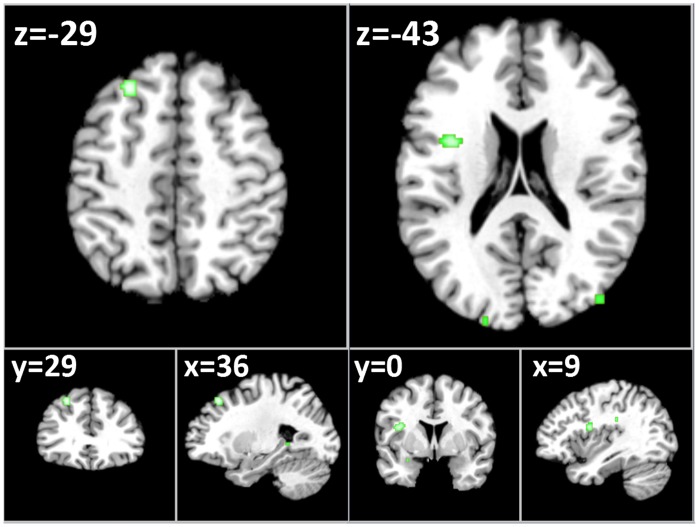
Axial (z), coronal (y), saggital (x) views of decreased activation to food images in fMRI studies of obese/overweight (versus healthy controls) - excluding Region of Interest Studies - FDR corrected, p<0.05 for multiple comparisons, cluster size >100 **mm^3^.** Left: reduced activation in the left dorsolateral prefrontal cortex (LDPFC); reduced activation in the left insular cortex.

A total of 6 of the 7 non-ROI studies contrasting food to non-food images contributed to two clusters of decreased activation in the left dorsolateral prefrontal cortex (DLPFC, x = −29, y = 29, z = 36) and left insular cortex (x = −43, y = 0, z = 9). See Table 2 and Figure 2.

### Secondary Meta-analysis: Food versus Non-food Stimuli

#### Increased activation in obese versus healthy controls

In response to food versus non-food stimuli (including taste, intake and smell of food versus non-food stimuli of the respective kind), a total of 8 of the 10 non-ROI studies contrasting food to non-food stimuli contributed to 7 clusters of increased activation after threshold correction (FDR) in the left lentiform nucleus (x = −16, y = 7, z = −8), right precentral gyrus (x = 52, y = −7, z = 23), left dorsomedial frontal gyrus (x = −4, y = 51, z = 24), right inferior frontal gyrus (x = 38, y = 17, z = −5), right parahippocampal gyrus (x = 21, y = −48, z = 1), left precentral gyrus (x = −49, y = −10, z = 30) and left anterior cingulate gyrus (x = −6, y = 5, z = 40). See Table 3.

**Table 3:** Locations of centered Talairach peak coordinates with significant ALE values for, respectively, *increased* (1) and decreased (2) activation in obese/overweight versus control subjects for all non-ROI fMRI studies looking at *food versus non-food stimuli* (including taste and food intake) in the meta-analysis.

Cluster^a^		Anatomical Label^b^		Peak voxel coordinates^c^			Cluster size	ALEvalue	Foci	
1. Obese/overweight>Normal-weight controls (8 contributing studies)										
				*x*	*y*	*z*	(mm^3^)	(×10^−2^)	*n*	*foci*
1	L. Lentiform Nucleus			−16	7	−8	1296	2.15	2	7
2	R. Precentral Gyrus			52	−2	23	1232	2.46	2	8
3	L. dorsomedial PFC			−4	51	24	768	2.17	2	5
4	R. Inferior Frontal Gyrus			38	17	−5	632	2.13	2	4
5	R. Parahippocampal Gyrus			21	−48	1	488	2.16	2	3
6	L. Precentral Gyrus			−49	−10	30	272	1.86	2	2
7	L. Cingulate Gyrus			−6	5	40	232	1.76	2	2
2. Normal-weight controls>obsese/overweight (7 contributing studies)										
			*x*		*y*	*z*	(mm^3^)	(×10^−2^)	*n*	*foci*
1. Normal-weight controls>Obese/overweight (7 contributing studies)										
1	L. DLPFC			−29	29	36	616	1.57	1	2
2	L. Insula			−43	−1	9	336	1.25	1	2

a ALE clusters at p<0.05 (FDR threshold correction for multiple comparisons, cluster size >100 mm^3^).

b L, left hemisphere, R, right hemisphere.

c Voxel coordinates are in Talairach space.

#### Decreased activation in all obese cases versus controls

A total of 7 of the 10 non-ROI studies contrasting food to non-food stimuli contributed to two clusters of decreased activation in the left dorsolateral prefrontal cortex (DLPFC, x = −29, y = 29, z = 36) and left insular cortex (x = −43, y = −1, z = 9). See Table 3.

## Discussion

According to significant cluster sizes, our most robust findings were of increased activation to food images in those who are obese or overweight, in the left dorsomedial prefrontal cortex, right precentral gyrus, right parahippocampal gyrus, right superior/inferior frontal gyrus and right anterior cingulate, regions associated with cognitive evaluation of salient stimuli, motor responses and explicit memory, suggesting that when people who are obese view images of food they think about the motivational reward of the food and previous experience of it. Additionally, we found preliminary significant evidence for reduced activation in the left dorsolateral prefrontal cortex (DLPFC) and left insular cortex, regions linked to cognitive control and interoceptive awareness respectively, which may be associated with reduced bodily responses to the anticipation of food (such that greater quantities of food need to be consumed to feel satisfied) and weakened attempts to control one’s appetite [20].

These collective findings are in line with contemporary views of functional brain abnormalities in those who overeat, and they provide some support for the addiction model of obesity, specifically, that there is reduced activation in brain regions linked to cognitive control. However, in contrast to the food addiction model of obesity, we did not observe significant differences in mesolimbic brain circuitry (e.g. dopamine-rich striatal activation), but rather that differences were observed in areas associated with somatosensory and memory processes. A lack of top-down control over appetite, combined with greater attention and memory resources aimed towards food stimuli, is likely to be a contributing factor in both obesity and addiction. However, it is plausible that being obese is, in contrast, more greatly associated with somatosensory deficits than mesolimbic reward neural circuitry malfunction, as in those with addiction. Somatosensory deficits may underlie a malfunctioning interoceptive awareness of hunger and satiety signals during the anticipation of food intake, such that greater quantities of food are consumed in those prone to obesity.

We observed increased activation in the left dorsomedial prefrontal, right precentral and right parahippocampal gyri in obese subjects, regions linked to attention, motor coordination, and explicit memory, and that are implicated in processes associated with the anticipation of food intake, particularly during perceived calorie deprivation [21]. Specifically, the dorsomedial prefrontal cortex has recently been shown to activate when those who are obesity prone view food images in the fasted state [22]. Activation of the parahippocampal gyrus has also been inversely linked to measures of satiety [23], suggesting that the parahippocampus might be involved in food-seeking behaviours when a person does not feel satiated. We also found increased activation in the right anterior cingulate cortex (ACC) in response to food images in those who are obese. Much fMRI evidence implicates the ACC in the pathology of obesity e.g. [24], [25], [26], particularly as a gateway between bottom-up mesolimbic reward responses and top-down cognitive control mechanisms [27], and as a general processor of conflict and error detection [28], [29], [30], e.g. between the desire to control appetite and the desire to eat. Additionally, activation of the ACC is associated with sensory processing of the body state during resting [20], [31], [32], which suggests that there may be abnormal processing of sensory information from the body in those who are obese. The ACC has been found to be thinner in obese compared to lean individuals [33]. Thus, it is plausible to observe a significant cluster of increased activation across studies of those who are obese, in the ACC, when evaluating a visual image of food.

The anatomical proximity between the ACC and DLPFC suggests that functional connectivity in prefrontal regions plays a large role in the perception of food images in people who are obese, e.g. [34]. It is likely that reward responses to food images are activated to varying degrees (e.g. modulated by level of anticipation and desire), but that successful restraint of appetitive responses is reflected by prefrontal systems [35]. An obese phenotype may be the result of a calculation in the brain between hypo-or hyperactive somatosensory (and in some cases mesolimbic) responses, and the necessity to control one’s behaviour (in relation to feeding). However, the relationship is complex, since thinking about eating food (following image presentation) versus consummation of food is likely associated with hyper- and hypoactivation in reward regions of the brain, respectively, in those who are prone to obesity. Indeed, a network incorporating the insula, parahippocampal gyrus and orbitofrontal cortex shows reduced activation after food consumption [36], and here we show increased activation in two of these regions to food anticipation (e.g. increased parahippocampal and mid-PFC). Furthermore, given that we do not observe significant differences in mesolimbic activation in our primary meta-analysis, but rather, differences in brain regions linked to body awareness and self-reference, it is plausible that different cognitive control mechanisms interact with somatosensory and reward responses to food. Thus, in these scenarios, prefrontal cortex systems likely calculate differing levels of cognitive modulation based on anticipation of how the food will be experienced in the body, supported by complex gene-environment interactions that flavour metabolic activity. As a result, caution must be exercised when inferring our neuroimaging data to the complex neurobiological mechanisms underlying obesity.

Many of the regions showing increased activity in obese/overweight compared to normal-weight controls have previously been linked to inhibitory control, such as the right middle and right inferior frontal gyri [37], involved in top-down control of appetitive processes. In view of the complex relationship between these processes, we also observed reduced left DLPFC but increased right IFG/DLPFC activation to food images across studies of overweight/obese people. The left DLPFC is associated with cognitive control, in some cases linked to appetite regulation, e.g. [38], the control of conflict, inhibition of impulsive behaviour and personality traits associated with behavioural inhibition [29], [39]. Some evidence points to a lateralization of DLPFC function, with the left DLPFC being associated with anticipatory cognitive control, the right DLPFC linked to control of immediate impulses and reducing attentional conflicts [40]. Disruption or reduced activation of right DLPFC by transcranial magnetic stimulation (TMS) increases immediate risk-taking behavior [41] and disrupts the ability to stop an immediate action that has been initiated in the stop-signal task (SST) [42], [43]. Thus, it could be that in obesity, there is a conflict between immediate and anticipatory cognitive control, with the latter motivating individuals to search for and think about food if this system is weakened. In our meta-analysis we found that activation across studies of the left DLPFC was reduced, but regions of the right PFC increased, suggesting reduced activation for anticipation of control, but increased activation for immediate control in response to food stimuli in the scanner. Among other personality traits, impulsivity has recently been found to be the strongest predictor of becoming overweight [44], and this trait has also been linked to reduced left DLPFC function [45]. In a study by Kozink et al., successful inhibition increased activation in the right DLPFC, suggesting that successful inhibitory behaviour during smoking abstinence requires increased attentional demand. Increased activation in the right IFG/DLPFC in obese subjects, as found in our meta-analyses, may therefore indicate that obese people also attempt to increase their recruitment of the right DLPFC when trying to suppress urges to consume food, particularly in response to images of food. Given the proposed similarities between obesity and addiction, e.g. [3], reduced left DLPFC activation might instead reflect vulnerability or malfunctioning in cognitive systems anticipating future food consumption, which is in line with a contemporary addiction neurobiological model of obesity [3].

It must also be borne in mind, that although these data go some way to supporting the addiction model of obesity, linking increased reward evaluation and decreased cognitive control to response to both all types of food stimuli as well as only images of food, there are still conflicting views concerning whether food addiction actually exists [4], [6], [7], [8]. For example, although many aspects of behaviour leading to obesity seem to mimic addictive behaviour, the neurobiological mechanisms underlying the development and metabolism defects of obesity are likely to be different, not least because food probably activates and alters the brain differently compared to addictive substances and behaviours such as cocaine, methamphetamine and gambling [8]. Additionally, we must be careful in labelling those who are obese as food addicts, given the inherent stigmatisation and blame afforded to a heightened perception of control (rather than biological factors) over one’s condition [4]. For example, only in our secondary (more inclusive but thus also more heterogeneous) but not our primary meta-analysis did we show significant differences between obese and healthy weight people in one mesolimbic reward region (the left lentiform nucleus). Our overall finding was rather, a reduced activation in a brain region that is associated with interoceptive awareness, that is, cognitive-emotional processing of the bodily state (e.g., appetitive signals from the body). This might suggest that people who are obese must consume more food to experience fully the interoceptive cues from the body, especially in relation to thinking about food when viewing food images, or that food images do not evoke as much interoceptive signalling in the brain as actual food consumption. However, in line with an addiction model, we also observed increased activation in prefrontal regions linked to cognitive evaluation of rewarding stimuli, and some decreased activation in a prefrontal region linked to cognitive control. Thus, while addiction and aberrant feeding behaviour likely have some dysfunctional brain networks in common, the contribution of brain networks to ‘food abuse’ [4] might lean, not towards the control of mesolimbic reward pathways, but to the modulation of interoceptive cues that determine one’s sense of hunger and satiety.

Interestingly, most of the regions we found to be activated in obese people, in response to either food images or all types of food stimuli, largely overlap with regions that have recently been found to be reduced in structural imaging studies in elderly overweight or obese subjects compared to normal-weight controls [46], [47]. In addition, reduced gray matter volume in prefrontal regions has also been associated with an increase in BMI at 1-year follow-up from baseline [48] and even in young obese adolescents with dis-inhibited eating, frontal brain regions appear to be reduced [49]. A correlation with brain activation and reduced brain volume, in left inferior frontal and left superior temporal gyri, has previously been reported for e.g. Alzheimer’s disease [50]. Such correlations for obesity between regions of increased activation in response to rewarding food stimuli and reduced brain volume could imply that the brain regions undergoing atrophy result in a higher load on these malfunctioning regions, much like the examples for the DLPFC given in the previous section, explaining the higher activation seen in these regions in our meta-analyses.

The results of our primary and secondary meta-analyses are also supported by alterations in neural response seen after Roux-en-Y gastric bypass (RYGB) surgery, typically performed on obese or morbidly obese subjects to achieve significant weight reductions (in the study by Ochner et al., a mean 1-month post-surgery BMI reduction of 5.6 kg/m^2^), where it was found that post-RYGB resulted in reduced activation in the lentiform nucleus, the posterior and anterior cingulate gyri and superior frontal, middle frontal and inferior frontal gyri in response to food images [51]. This data supports the notion that the regions found in our meta-analyses are a result of requirement for inhibitory control in obese compared to normal-weight subjects.

### Strengths and Limitations

There are some limitations to our ALE meta-analyses. Only a limited number of studies met our inclusion criteria, which is why we chose to conduct two separate meta-analyses, analysing the neural response in obese compared to normal-weight subjects to 1) only food images (primary meta-analysis), and 2) all food stimuli including odor, taste, ingestion (secondary meta-analysis), versus non-food images or stimuli, respectively. However, we found that the results were largely unaffected by adding the three studies using food stimuli such as intake, taste or odors of foods, and thus increasing the total number of subjects in the meta-analysis from 200 to 255 and the number of foci from 121 to 184. This implies that the findings of our primary meta-analysis, looking specifically at studies employing images as food stimuli, were fairly robust. Although, in our meta-analyses we found that activation, as opposed to deactivation was the most robust when considering the number of contributing studies and foci.

The ALE method does not take several important statistical factors into account. These include BOLD signal strength, cluster size and statistical significance of each included foci. Future updates of this useful tool for meta-analysing fMRI data are likely to incorporate these current limitations, although at present many other reviews have successfully used ALE to meta-analyse fMRI data, e.g. [11], [12], [13], [14], [15]. Additionally, many studies conduct both ROI and whole-brain analyses, e.g. by setting a different significance threshold for a set of a priori known relevant brain regions. In these cases it is oftentimes not specified in the results section whether results were from either ROI or whole-brain analyses, making meta-analysis more difficult. Furthermore, given the variability of food stimuli between studies, it was not possible to conduct a separate analysis of only consummatory stimuli, which is likely important, in order to pinpoint different brain circuits. Finally, the inherent variability of fMRI studies makes it difficult to draw definitive conclusions about activation likelihoods. However, combined with the wealth of qualitative reviews on fMRI findings in obesity, this first attempt at quantitatively reviewing the data will strengthen our knowledge of the neural correlates potentially underlying responses to images of food in those who are obese or overweight.

### Conclusions

Thinking about eating food shown in images, in those who are obese or overweight coincides with an increased neural activation in brain regions associated with detection, remembering and monitoring of desirable foodstuff, in conjunction with a reduced cognitive control and interoceptive brain response. A weakened or malfunctioning cognitive control system perhaps derived from a preponderance to anticipate the consumption of appetising food may underlie the obese-risk brain. These data support an addiction model of obesity to the extent that a weakened cognitive control system contributes to an inability to restrain appetite. However, the food addiction model implicates an aberrant dopaminergic mesolimbic response in obesity, for which we did not observe strong evidence here, but rather we found differences in interoceptive responses were more apparent. Thus, cognitive evaluation and insufficient cognitive control associated with the anticipation of bodily sensations during food ingestion is perhaps more important in the generation and maintenance of obesity, than the cognitive modulation of mesolimbic reward responses to food per se. Cognitive therapies aiming to reduce cognitive biases for anticipating food ingestion, while strengthening cognitions pertaining to the restraint of appetite, will likely help to lower obesity rates in Western societies that are abundant in food advertising and readily available appetising food.

## Supporting Information

Figure S1 **PRISMA Flowchart.**
(DOC)

Table S1 **List of the studies and reason for exclusion for those that were initially excluded from the actual meta-analyses.**
(XLSX)

Table S2 **List of the studies and reason for exclusion for those that were excluded at a secondary stage from the actual meta-analyses.**
(XLSX)

Table S3 **Checklist for PRISMA items.**
(DOC)
