# Supplementary material for: Increased Prefrontal and Parahippocampal Activation with Reduced Dorsolateral Prefrontal and Insular Cortex Activation to Food Images in Obesity: A Meta-Analysis of fMRI Studies
Source: PLoS One. 2013 Apr 10;8(4):e60393. doi: 10.1371/journal.pone.0060393 (PMC3622693; doi:10.1371/journal.pone.0060393)
Supplement: Figure S1 — PRISMA Flowchart. (DOC) [file pone.0060393.s001.doc]

**Identification**

**Screening**

**Eligibility**

**Included**

3790 records identified through database searching

483 of records screened

407 records excluded

76 full-text articles assessed for eligibility

69 full-text articles excluded (66 fully excluded):

40: Other eating disorders besides obesity (e.g. Anorexia Nervosa, Bulimia Nervosa, Prader Willi Syndrome)

15: Only Region of Interest analyses (3 of these also had WB analyses; this WB data was included)

14: No case-control fMRI data

10 studies included in quantitative synthesis (meta-analysis):

7/10 studies used for primary meta-analysis (food *images*)

10/10 studies used for secondary meta-analysis (food *stimuli*)
